# 132 Detailed Characterization of a Novel Human Ex Vivo Model for Burn Injuries

**DOI:** 10.1093/jbcr/irae036.131

**Published:** 2024-04-17

**Authors:** Petra Kotzbeck, Elisabeth Hofmann, Ines Foessl, Julia Fink, Anita Eberl, Christoph Magnes, Dagmar Kolb, Thomas Birngruber, Martin Funk, Lars-Peter Kamolz

**Affiliations:** Joanneum Research - COREMED, Graz, Steiermark; JOANNEUM RESEARCH Forschungsgesellschaft mbH, COREMED, Graz, Steiermark; Medical University Graz, Division of Endocrinology and Diabetology, Graz, Steiermark; JOANNEUM RESEARCH Forschungsgesellschaft mbH, HEALTH, Graz, Steiermark; Medical University Graz, Core Facility Ultrastructure Analysis, Graz, Steiermark; EVOMEDIS GmbH, Graz, Steiermark; Medical University Graz, Graz, Steiermark; Joanneum Research - COREMED, Graz, Steiermark; JOANNEUM RESEARCH Forschungsgesellschaft mbH, COREMED, Graz, Steiermark; Medical University Graz, Division of Endocrinology and Diabetology, Graz, Steiermark; JOANNEUM RESEARCH Forschungsgesellschaft mbH, HEALTH, Graz, Steiermark; Medical University Graz, Core Facility Ultrastructure Analysis, Graz, Steiermark; EVOMEDIS GmbH, Graz, Steiermark; Medical University Graz, Graz, Steiermark; Joanneum Research - COREMED, Graz, Steiermark; JOANNEUM RESEARCH Forschungsgesellschaft mbH, COREMED, Graz, Steiermark; Medical University Graz, Division of Endocrinology and Diabetology, Graz, Steiermark; JOANNEUM RESEARCH Forschungsgesellschaft mbH, HEALTH, Graz, Steiermark; Medical University Graz, Core Facility Ultrastructure Analysis, Graz, Steiermark; EVOMEDIS GmbH, Graz, Steiermark; Medical University Graz, Graz, Steiermark; Joanneum Research - COREMED, Graz, Steiermark; JOANNEUM RESEARCH Forschungsgesellschaft mbH, COREMED, Graz, Steiermark; Medical University Graz, Division of Endocrinology and Diabetology, Graz, Steiermark; JOANNEUM RESEARCH Forschungsgesellschaft mbH, HEALTH, Graz, Steiermark; Medical University Graz, Core Facility Ultrastructure Analysis, Graz, Steiermark; EVOMEDIS GmbH, Graz, Steiermark; Medical University Graz, Graz, Steiermark; Joanneum Research - COREMED, Graz, Steiermark; JOANNEUM RESEARCH Forschungsgesellschaft mbH, COREMED, Graz, Steiermark; Medical University Graz, Division of Endocrinology and Diabetology, Graz, Steiermark; JOANNEUM RESEARCH Forschungsgesellschaft mbH, HEALTH, Graz, Steiermark; Medical University Graz, Core Facility Ultrastructure Analysis, Graz, Steiermark; EVOMEDIS GmbH, Graz, Steiermark; Medical University Graz, Graz, Steiermark; Joanneum Research - COREMED, Graz, Steiermark; JOANNEUM RESEARCH Forschungsgesellschaft mbH, COREMED, Graz, Steiermark; Medical University Graz, Division of Endocrinology and Diabetology, Graz, Steiermark; JOANNEUM RESEARCH Forschungsgesellschaft mbH, HEALTH, Graz, Steiermark; Medical University Graz, Core Facility Ultrastructure Analysis, Graz, Steiermark; EVOMEDIS GmbH, Graz, Steiermark; Medical University Graz, Graz, Steiermark; Joanneum Research - COREMED, Graz, Steiermark; JOANNEUM RESEARCH Forschungsgesellschaft mbH, COREMED, Graz, Steiermark; Medical University Graz, Division of Endocrinology and Diabetology, Graz, Steiermark; JOANNEUM RESEARCH Forschungsgesellschaft mbH, HEALTH, Graz, Steiermark; Medical University Graz, Core Facility Ultrastructure Analysis, Graz, Steiermark; EVOMEDIS GmbH, Graz, Steiermark; Medical University Graz, Graz, Steiermark; Joanneum Research - COREMED, Graz, Steiermark; JOANNEUM RESEARCH Forschungsgesellschaft mbH, COREMED, Graz, Steiermark; Medical University Graz, Division of Endocrinology and Diabetology, Graz, Steiermark; JOANNEUM RESEARCH Forschungsgesellschaft mbH, HEALTH, Graz, Steiermark; Medical University Graz, Core Facility Ultrastructure Analysis, Graz, Steiermark; EVOMEDIS GmbH, Graz, Steiermark; Medical University Graz, Graz, Steiermark; Joanneum Research - COREMED, Graz, Steiermark; JOANNEUM RESEARCH Forschungsgesellschaft mbH, COREMED, Graz, Steiermark; Medical University Graz, Division of Endocrinology and Diabetology, Graz, Steiermark; JOANNEUM RESEARCH Forschungsgesellschaft mbH, HEALTH, Graz, Steiermark; Medical University Graz, Core Facility Ultrastructure Analysis, Graz, Steiermark; EVOMEDIS GmbH, Graz, Steiermark; Medical University Graz, Graz, Steiermark; Joanneum Research - COREMED, Graz, Steiermark; JOANNEUM RESEARCH Forschungsgesellschaft mbH, COREMED, Graz, Steiermark; Medical University Graz, Division of Endocrinology and Diabetology, Graz, Steiermark; JOANNEUM RESEARCH Forschungsgesellschaft mbH, HEALTH, Graz, Steiermark; Medical University Graz, Core Facility Ultrastructure Analysis, Graz, Steiermark; EVOMEDIS GmbH, Graz, Steiermark; Medical University Graz, Graz, Steiermark

## Abstract

**Introduction:**

Burn injuries belong to the most common soft tissue injuries and often result in extensive trauma but are also associated with cases of major emergency such as sepsis or systemic inflammatory response syndrome. The early phase after burn injury is critical and decides about later potential complications in wound healing. Burn injuries activate numerous processes, including heat shock, inflammation and tissue regeneration responses and thereby promote the release of cytokines and other signalling molecules such as miRNAs and metabolites. Despite extensive research, skin tissue reactions in the early phases after burn injuries still need to be investigated in more detail. Therefore, reliable burn models are needed to elucidate the exact sequence of events during the healing process and to monitor potential biomarkers that could give information about treatment success.

**Methods:**

We induced contact burns on fresh human abdominal skin explants that were resected during abdominoplasty. Gene and miRNA expression patterns, cytokine production profiles of key mediators such as IL8 and IL6 and skin ultrastructure were analysed for 24 hours after the burn injury. Additionally, we used open flow microperfusion (OFM), a sampling technique that allows time and location dependent collection of dermal interstitial fluid (dISF), to also monitor the release of specific miRNAs, cytokines and metabolites.

**Results:**

In burn injuries, we found significant changes in gene and miRNA expression as soon as one hour after burn injury. Inflammatory genes such as IL8 were significantly up-regulated whereas miRNAs were systematically down-regulated. miRNAs were actively released and mobilized into the dISF, while miR-497-5p could be identified stably downregulated in tissue and dISF in the early phase after a burn injury. Metabolome analysis of dISF showed a striking metabolic shift in burn injuries, linking burn injury to increased amino acid and glucose turnover in wounds.

**Conclusions:**

By using this ex vivo human skin model for burn injuries we were able to study the immediate early responses to burns for up to 24 h. We found that miR-497-5p could serve as potential biomarker to assess burn severity since it is not only down-regulated in skin but also mobilized into the dISF after burn injury. Furthermore, we found distinct metabolite signatures after burn injury.

**Applicability of Research to Practice:**

Identification and research on potential biomarkers to assess burn severity and treatment success.

